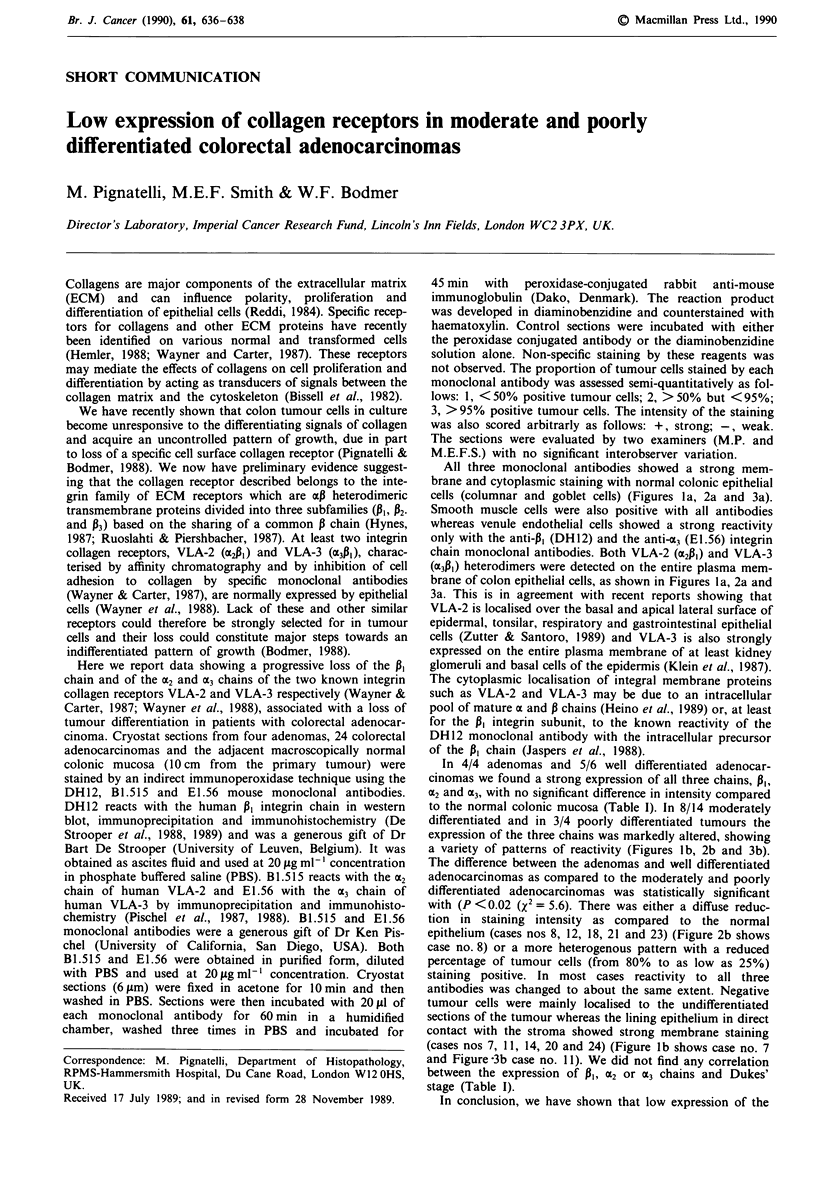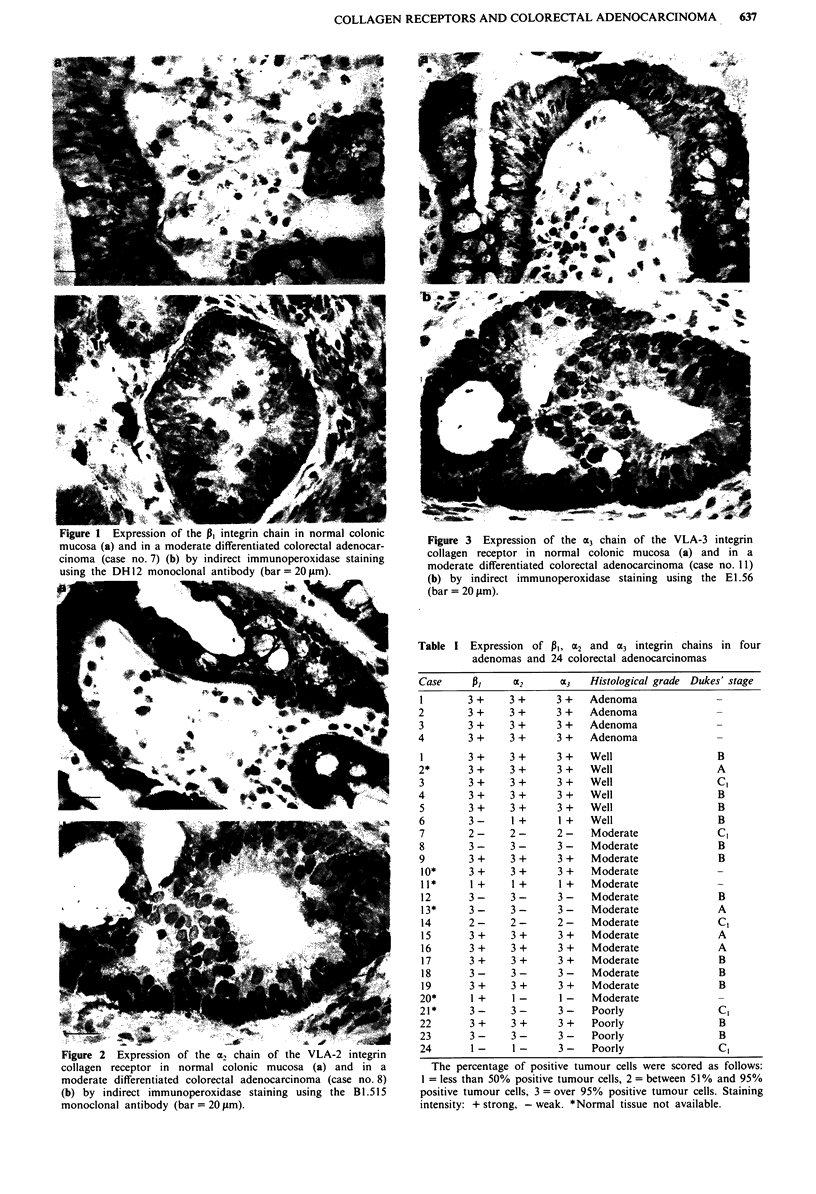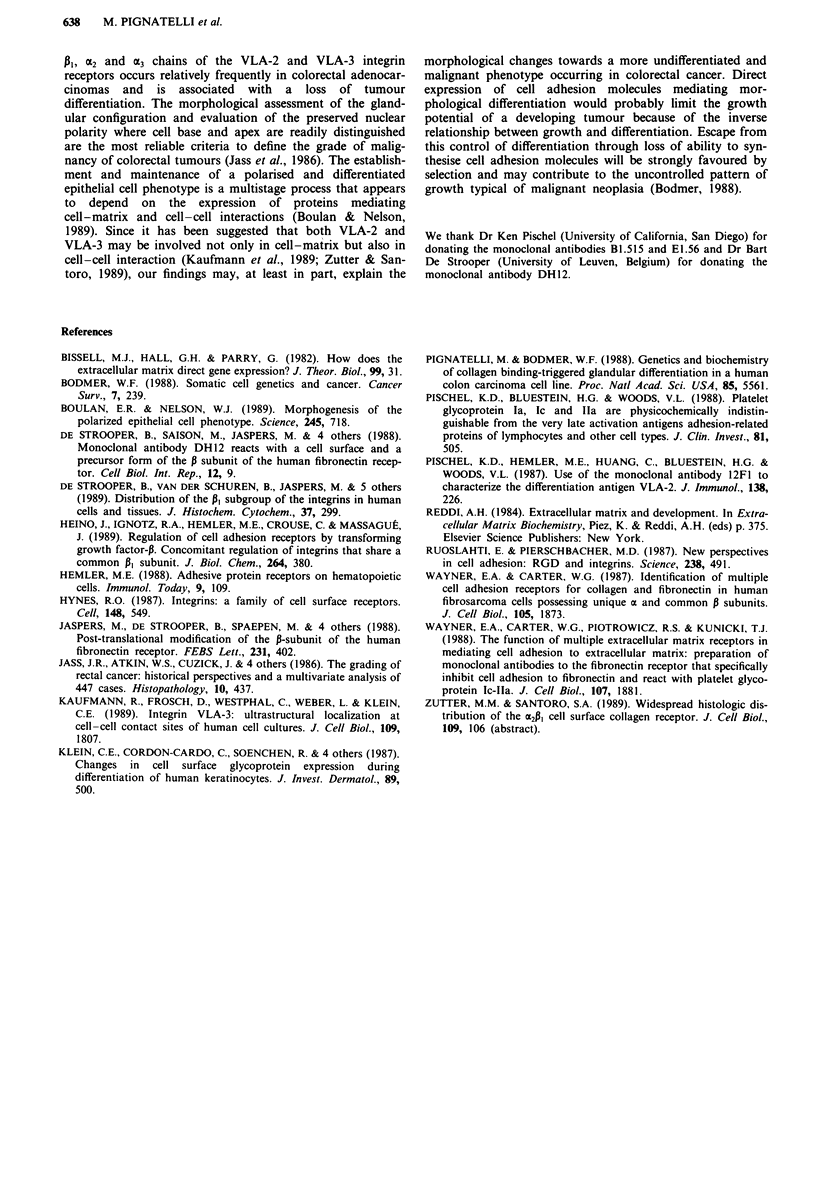# Low expression of collagen receptors in moderate and poorly differentiated colorectal adenocarcinomas.

**DOI:** 10.1038/bjc.1990.141

**Published:** 1990-04

**Authors:** M. Pignatelli, M. E. Smith, W. F. Bodmer

**Affiliations:** Director's Laboratory, Imperial Cancer Research Fund, London, UK.

## Abstract

**Images:**


Br. J. Cancer (1990), 61, 636-638 © Macmillan Press Ltd., 1990

SHORT COMMUNICATION

### Low expression of collagen receptors in moderate and poorly differentiated colorectal adenocarcinomas

M. Pignatelli, M.E.F. Smith & W.F. Bodmer

*Director's Laboratory, Imperial Cancer Research Fund, Lincoln's Inn Fields, London WC2 3PX, UK.*

Collagens are major components of the extracellular matrix (ECM) and can influence polarity, proliferation and differentiation of epithelial cells (Reddi, 1984). Specific receptors for collagens and other ECM proteins have recently been identified on various normal and transformed cells (Hemler, 1988; Wayner and Carter, 1987). These receptors may mediate the effects of collagens on cell proliferation and differentiation by acting as transducers of signals between the collagen matrix and the cytoskeleton (Bissell *et al.*, 1982).

We have recently shown that colon tumour cells in culture become unresponsive to the differentiating signals of collagen and acquire an uncontrolled pattern of growth, due in part to loss of a specific cell surface collagen receptor (Pignatelli & Bodmer, 1988). We now have preliminary evidence suggesting that the collagen receptor described belongs to the integrin family of ECM receptors which are $\alpha\beta$ heterodimeric transmembrane proteins divided into three subfamilies ($\beta_1$, $\beta_2$. and $\beta_3$) based on the sharing of a common $\beta$ chain (Hynes, 1987; Ruoslahti & Piershbacher, 1987). At least two integrin collagen receptors, VLA-2 ($\alpha_2\beta_1$) and VLA-3 ($\alpha_3\beta_1$), characterised by affinity chromatography and by inhibition of cell adhesion to collagen by specific monoclonal antibodies (Wayner & Carter, 1987), are normally expressed by epithelial cells (Wayner *et al.*, 1988). Lack of these and other similar receptors could therefore be strongly selected for in tumour cells and their loss could constitute major steps towards an indifferentiated pattern of growth (Bodmer, 1988).

Here we report data showing a progressive loss of the $\beta_1$ chain and of the $\alpha_2$ and $\alpha_3$ chains of the two known integrin collagen receptors VLA-2 and VLA-3 respectively (Wayner & Carter, 1987; Wayner *et al.*, 1988), associated with a loss of tumour differentiation in patients with colorectal adenocarcinoma. Cryostat sections from four adenomas, 24 colorectal adenocarcinomas and the adjacent macroscopically normal colonic mucosa (10 cm from the primary tumour) were stained by an indirect immunoperoxidase technique using the DH12, B1.515 and E1.56 mouse monoclonal antibodies. DH12 reacts with the human $\beta_1$ integrin chain in western blot, immunoprecipitation and immunohistochemistry (De Strooper *et al.*, 1988, 1989) and was a generous gift of Dr Bart De Strooper (University of Leuven, Belgium). It was obtained as ascites fluid and used at 20 $\mu$g ml$^{-1}$ concentration in phosphate buffered saline (PBS). B1.515 reacts with the $\alpha_2$ chain of human VLA-2 and E1.56 with the $\alpha_3$ chain of human VLA-3 by immunoprecipitation and immunohistochemistry (Pischel *et al.*, 1987, 1988). B1.515 and E1.56 monoclonal antibodies were a generous gift of Dr Ken Pischel (University of California, San Diego, USA). Both B1.515 and E1.56 were obtained in purified form, diluted with PBS and used at 20 $\mu$g ml$^{-1}$ concentration. Cryostat sections (6 $\mu$m) were fixed in acetone for 10 min and then washed in PBS. Sections were then incubated with 20 $\mu$l of each monoclonal antibody for 60 min in a humidified chamber, washed three times in PBS and incubated for 45 min with peroxidase-conjugated rabbit anti-mouse immunoglobulin (Dako, Denmark). The reaction product was developed in diaminobenzidine and counterstained with haematoxylin. Control sections were incubated with either the peroxidase conjugated antibody or the diaminobenzidine solution alone. Non-specific staining by these reagents was not observed. The proportion of tumour cells stained by each monoclonal antibody was assessed semi-quantitatively as follows: 1, $<50\%$ positive tumour cells; 2, $>50\%$ but $<95\%$; 3, $>95\%$ positive tumour cells. The intensity of the staining was also scored arbitrarily as follows: +, strong; −, weak. The sections were evaluated by two examiners (M.P. and M.E.F.S.) with no significant interobserver variation.

All three monoclonal antibodies showed a strong membrane and cytoplasmic staining with normal colonic epithelial cells (columnar and goblet cells) (Figures 1a, 2a and 3a). Smooth muscle cells were also positive with all antibodies whereas venule endothelial cells showed a strong reactivity only with the anti-$\beta_1$ (DH12) and the anti-$\alpha_3$ (E1.56) integrin chain monoclonal antibodies. Both VLA-2 ($\alpha_2\beta_1$) and VLA-3 ($\alpha_3\beta_1$) heterodimers were detected on the entire plasma membrane of colon epithelial cells, as shown in Figures 1a, 2a and 3a. This is in agreement with recent reports showing that VLA-2 is localised over the basal and apical lateral surface of epidermal, tonsilar, respiratory and gastrointestinal epithelial cells (Zutter & Santoro, 1989) and VLA-3 is also strongly expressed on the entire plasma membrane of at least kidney glomeruli and basal cells of the epidermis (Klein *et al.*, 1987). The cytoplasmic localisation of integral membrane proteins such as VLA-2 and VLA-3 may be due to an intracellular pool of mature $\alpha$ and $\beta$ chains (Heino *et al.*, 1989) or, at least for the $\beta_1$ integrin subunit, to the known reactivity of the DH12 monoclonal antibody with the intracellular precursor of the $\beta_1$ chain (Jaspers *et al.*, 1988).

In 4/4 adenomas and 5/6 well differentiated adenocarcinomas we found a strong expression of all three chains, $\beta_1$, $\alpha_2$ and $\alpha_3$, with no significant difference in intensity compared to the normal colonic mucosa (Table I). In 8/14 moderately differentiated and in 3/4 poorly differentiated tumours the expression of the three chains was markedly altered, showing a variety of patterns of reactivity (Figures 1b, 2b and 3b). The difference between the adenomas and well differentiated adenocarcinomas as compared to the moderately and poorly differentiated adenocarcinomas was statistically significant with ($P<0.02$ ($\chi^2 = 5.6$). There was either a diffuse reduction in staining intensity as compared to the normal epithelium (cases nos 8, 12, 18, 21 and 23) (Figure 2b shows case no. 8) or a more heterogenous pattern with a reduced percentage of tumour cells (from 80% to as low as 25%) staining positive. In most cases reactivity to all three antibodies was changed to about the same extent. Negative tumour cells were mainly localised to the undifferentiated sections of the tumour whereas the lining epithelium in direct contact with the stroma showed strong membrane staining (cases nos 7, 11, 14, 20 and 24) (Figure 1b shows case no. 7 and Figure 3b case no. 11). We did not find any correlation between the expression of $\beta_1$, $\alpha_2$ or $\alpha_3$ chains and Dukes' stage (Table I).

In conclusion, we have shown that low expression of the

Correspondence: M. Pignatelli, Department of Histopathology, RPMS-Hammersmith Hospital, Du Cane Road, London W12 0HS, UK.
Received 17 July 1989; and in revised form 28 November 1989.

**Figure 1** Expression of the $\beta_1$ integrin chain in normal colonic mucosa (**a**) and in a moderate differentiated colorectal adenocarcinoma (case no. 7) (**b**) by indirect immunoperoxidase staining using the DH12 monoclonal antibody (bar = 20 μm).

**Figure 2** Expression of the $\alpha_2$ chain of the VLA-2 integrin collagen receptor in normal colonic mucosa (**a**) and in a moderate differentiated colorectal adenocarcinoma (case no. 8) (**b**) by indirect immunoperoxidase staining using the B1.515 monoclonal antibody (bar = 20 μm).

**Figure 3** Expression of the $\alpha_3$ chain of the VLA-3 integrin collagen receptor in normal colonic mucosa (**a**) and in a moderate differentiated colorectal adenocarcinoma (case no. 11) (**b**) by indirect immunoperoxidase staining using the E1.56 (bar = 20 μm).

**Table I** Expression of $\beta_1$, $\alpha_2$ and $\alpha_3$ integrin chains in four adenomas and 24 colorectal adenocarcinomas

| *Case* | $\beta_1$ | $\alpha_2$ | $\alpha_3$ | *Histological grade* | *Dukes' stage* |
|---|---|---|---|---|---|
| 1 | 3+ | 3+ | 3+ | Adenoma | – |
| 2 | 3+ | 3+ | 3+ | Adenoma | – |
| 3 | 3+ | 3+ | 3+ | Adenoma | – |
| 4 | 3+ | 3+ | 3+ | Adenoma | – |
| 1 | 3+ | 3+ | 3+ | Well | B |
| 2* | 3+ | 3+ | 3+ | Well | A |
| 3 | 3+ | 3+ | 3+ | Well | $C_1$ |
| 4 | 3+ | 3+ | 3+ | Well | B |
| 5 | 3+ | 3+ | 3+ | Well | B |
| 6 | 3– | 1+ | 1+ | Well | B |
| 7 | 2– | 2– | 2– | Moderate | $C_1$ |
| 8 | 3– | 3– | 3– | Moderate | B |
| 9 | 3+ | 3+ | 3+ | Moderate | B |
| 10* | 3+ | 3+ | 3+ | Moderate | – |
| 11* | 1+ | 1+ | 1+ | Moderate | – |
| 12 | 3– | 3– | 3– | Moderate | B |
| 13* | 3– | 3– | 3– | Moderate | A |
| 14 | 2– | 2– | 2– | Moderate | $C_1$ |
| 15 | 3+ | 3+ | 3+ | Moderate | A |
| 16 | 3+ | 3+ | 3+ | Moderate | A |
| 17 | 3+ | 3+ | 3+ | Moderate | B |
| 18 | 3– | 3– | 3– | Moderate | B |
| 19 | 3+ | 3+ | 3+ | Moderate | B |
| 20* | 1+ | 1– | 1– | Moderate | – |
| 21* | 3– | 3– | 3– | Poorly | $C_1$ |
| 22 | 3+ | 3+ | 3+ | Poorly | B |
| 23 | 3– | 3– | 3– | Poorly | B |
| 24 | 1– | 1– | 3– | Poorly | $C_1$ |

The percentage of positive tumour cells were scored as follows: 1 = less than 50% positive tumour cells, 2 = between 51% and 95% positive tumour cells, 3 = over 95% positive tumour cells. Staining intensity: + strong, – weak. *Normal tissue not available.

$\beta_1$, $\alpha_2$ and $\alpha_3$ chains of the VLA-2 and VLA-3 integrin receptors occurs relatively frequently in colorectal adenocarcinomas and is associated with a loss of tumour differentiation. The morphological assessment of the glandular configuration and evaluation of the preserved nuclear polarity where cell base and apex are readily distinguished are the most reliable criteria to define the grade of malignancy of colorectal tumours (Jass *et al.*, 1986). The establishment and maintenance of a polarised and differentiated epithelial cell phenotype is a multistage process that appears to depend on the expression of proteins mediating cell–matrix and cell–cell interactions (Boulan & Nelson, 1989). Since it has been suggested that both VLA-2 and VLA-3 may be involved not only in cell–matrix but also in cell–cell interaction (Kaufmann *et al.*, 1989; Zutter & Santoro, 1989), our findings may, at least in part, explain the morphological changes towards a more undifferentiated and malignant phenotype occurring in colorectal cancer. Direct expression of cell adhesion molecules mediating morphological differentiation would probably limit the growth potential of a developing tumour because of the inverse relationship between growth and differentiation. Escape from this control of differentiation through loss of ability to synthesise cell adhesion molecules will be strongly favoured by selection and may contribute to the uncontrolled pattern of growth typical of malignant neoplasia (Bodmer, 1988).

We thank Dr Ken Pischel (University of California, San Diego) for donating the monoclonal antibodies B1.515 and E1.56 and Dr Bart De Strooper (University of Leuven, Belgium) for donating the monoclonal antibody DH12.

### References

BISSELL, M.J., HALL, G.H. & PARRY, G. (1982). How does the extracellular matrix direct gene expression? *J. Theor. Biol.*, **99**, 31.

BODMER, W.F. (1988). Somatic cell genetics and cancer. *Cancer Surv.*, **7**, 239.

BOULAN, E.R. & NELSON, W.J. (1989). Morphogenesis of the polarized epithelial cell phenotype. *Science*, **245**, 718.

DE STROOPER, B., SAISON, M., JASPERS, M. & 4 others (1988). Monoclonal antibody DH12 reacts with a cell surface and a precursor form of the $\beta$ subunit of the human fibronectin receptor. *Cell Biol. Int. Rep.*, **12**, 9.

DE STROOPER, B., VAN DER SCHUREN, B., JASPERS, M. & 5 others (1989). Distribution of the $\beta_1$ subgroup of the integrins in human cells and tissues. *J. Histochem. Cytochem.*, **37**, 299.

HEINO, J., IGNOTZ, R.A., HEMLER, M.E., CROUSE, C. & MASSAGUÉ, J. (1989). Regulation of cell adhesion receptors by transforming growth factor-$\beta$. Concomitant regulation of integrins that share a common $\beta_1$ subunit. *J. Biol. Chem.*, **264**, 380.

HEMLER, M.E. (1988). Adhesive protein receptors on hematopoietic cells. *Immunol. Today*, **9**, 109.

HYNES, R.O. (1987). Integrins: a family of cell surface receptors. *Cell*, **148**, 549.

JASPERS, M., DE STROOPER, B., SPAEPEN, M. & 4 others (1988). Post-translational modification of the $\beta$-subunit of the human fibronectin receptor. *FEBS Lett.*, **231**, 402.

JASS, J.R., ATKIN, W.S., CUZICK, J. & 4 others (1986). The grading of rectal cancer: historical perspectives and a multivariate analysis of 447 cases. *Histopathology*, **10**, 437.

KAUFMANN, R., FROSCH, D., WESTPHAL, C., WEBER, L. & KLEIN, C.E. (1989). Integrin VLA-3: ultrastructural localization at cell–cell contact sites of human cell cultures. *J. Cell Biol.*, **109**, 1807.

KLEIN, C.E., CORDON-CARDO, C., SOENCHEN, R. & 4 others (1987). Changes in cell surface glycoprotein expression during differentiation of human keratinocytes. *J. Invest. Dermatol.*, **89**, 500.

PIGNATELLI, M. & BODMER, W.F. (1988). Genetics and biochemistry of collagen binding-triggered glandular differentiation in a human colon carcinoma cell line. *Proc. Natl Acad. Sci. USA*, **85**, 5561.

PISCHEL, K.D., BLUESTEIN, H.G. & WOODS, V.L. (1988). Platelet glycoprotein Ia, Ic and IIa are physicochemically indistinguishable from the very late activation antigens adhesion-related proteins of lymphocytes and other cell types. *J. Clin. Invest.*, **81**, 505.

PISCHEL, K.D., HEMLER, M.E., HUANG, C., BLUESTEIN, H.G. & WOODS, V.L. (1987). Use of the monoclonal antibody 12F1 to characterize the differentiation antigen VLA-2. *J. Immunol.*, **138**, 226.

REDDI, A.H. (1984). Extracellular matrix and development. In *Extracellular Matrix Biochemistry*, Piez, K. & Reddi, A.H. (eds) p. 375. Elsevier Science Publishers: New York.

RUOSLAHTI, E. & PIERSCHBACHER, M.D. (1987). New perspectives in cell adhesion: RGD and integrins. *Science*, **238**, 491.

WAYNER, E.A. & CARTER, W.G. (1987). Identification of multiple cell adhesion receptors for collagen and fibronectin in human fibrosarcoma cells possessing unique $\alpha$ and common $\beta$ subunits. *J. Cell Biol.*, **105**, 1873.

WAYNER, E.A., CARTER, W.G., PIOTROWICZ, R.S. & KUNICKI, T.J. (1988). The function of multiple extracellular matrix receptors in mediating cell adhesion to extracellular matrix: preparation of monoclonal antibodies to the fibronectin receptor that specifically inhibit cell adhesion to fibronectin and react with platelet glycoprotein Ic-IIa. *J. Cell Biol.*, **107**, 1881.

ZUTTER, M.M. & SANTORO, S.A. (1989). Widespread histologic distribution of the $\alpha_2\beta_1$ cell surface collagen receptor. *J. Cell Biol.*, **109**, 106 (abstract).